# Recovery and protection of photo‐damaged hairs with keratin‐encapsulated cationic liposomes

**DOI:** 10.1111/ics.13080

**Published:** 2025-06-06

**Authors:** Hoai‐Thuong Duc Bui, Su Ji Kim, Jihui Jang, Jun Bae Lee, Hyuk Sang Yoo

**Affiliations:** ^1^ Department of Biomedical Science Kangwon National University Chuncheon Korea; ^2^ Innovation Lab Cosmax R&I Center Seongnam‐si Korea

**Keywords:** cationic liposomes, hair recovery, keratin, penetration enhancement, photo‐damaged hair

## Abstract

**Objective:**

Daily sunlight exposure impacts the structural integrity and chemical composition of hair in both reversible and permanent manners. Although keratin‐based treatments have been popularly used to repair damaged hair, their effects in the recovery of the hair damage caused by UV light have not been well studied. Moreover, limitations related to keratin penetration depth and duration of effect persist. Encapsulating active ingredients in liposomes has been shown to enhance their stability, bioavailability and permeability. Thus, we aimed to design cationic liposomes encapsulated with keratin to enhance the recovery effects of photo‐damaged hair.

**Methods:**

We prepared keratin‐incorporated cationic liposomes (KLs) via high‐pressure homogenization and assessed their physical properties via hydrodynamic size, zeta potential, electron microscopy and X‐ray diffraction analyses. The physical and chemical damage level of hair samples before and after treatment, such as hair smoothness, protein degradation and lipid peroxidation, were subsequently investigated using electron microscopy and different spectroscopies.

**Results:**

The results indicated that the cationic liposomes fabricated by high‐pressure homogenization method had combined unilamellar and multilamellar structures. Compared with the keratin solution, the KL suspension significantly improved the permeation of keratin into the cortex during a 24‐h incubation. Moreover, 24 and 48‐h UV‐exposed hairs showed enhanced recovery when incubated with keratin liposomes for 24 h, as evidenced by the observation of a smoother hair surface using electron microscopy. We observed that treatment with keratin liposomes significantly reduced protein denaturation and lipid peroxidation in the photo‐damaged hairs.

**Conclusion:**

We anticipate that cationic liposome‐assisted keratin delivery may serve as an effective method for restoring photo‐damaged hair in both the physical and chemical dimensions.

## Graphical abstract

Homogeneous and highly stable cationic liposomes encapsulated with keratin were fabricated using the high‐pressure homogenization method, which could improve the adherence of keratin on the hair cuticle and its penetration into hair fibres, thereby effectively enhancing the recovery of photo‐damaged hair.

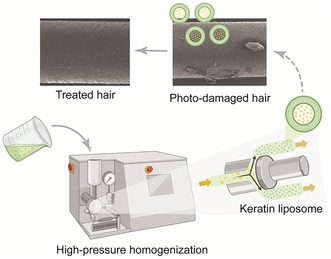

## Résumé

**Objectif:**

L'exposition quotidienne au soleil a un impact sur l'intégrité structurelle et la composition chimique des cheveux, de manière réversible ou permanente. Bien que les traitements à base de kératine aient été largement utilisés pour réparer les cheveux abîmés, leurs effets sur la réparation des dommages causés par la lumière UV n'ont pas été bien étudiés. En outre, les limitations liées à la profondeur de pénétration de la kératine et à la durée de l'effet persistent. Il a été démontré que l'encapsulation de principes actifs dans les liposomes améliore leur stabilité, leur biodisponibilité et leur perméabilité. Ainsi, nous avons cherché à concevoir des liposomes cationiques encapsulés dans de la kératine pour améliorer les effets de récupération des cheveux photo‐endommagés.

**Méthodes:**

Nous avons préparé des liposomes cationiques incorporés à la kératine (KL) par homogénéisation à haute pression et évalué leurs propriétés physiques par l'analyse de la taille hydrodynamique, du potentiel zêta, de la microscopie électronique et de la diffraction des rayons X. Le niveau de dommages physiques et chimiques des échantillons de cheveux avant et après le traitement, tels que la douceur des cheveux, la dégradation des protéines et la peroxydation lipidique, a ensuite été étudié à l'aide de microscopie électronique et de différentes spectroscopies.

**Résultats:**

Les résultats indiquent que les liposomes cationiques fabriqués par la méthode d'homogénéisation à haute pression ont des structures unilamellaires et multilamellaires combinées. Par rapport à la solution de kératine, la suspension de KL a amélioré de manière significative la perméation de la kératine dans le cortex pendant une incubation de 24 heures. En outre, les cheveux exposés aux UV pendant 24 et 48 heures se sont mieux rétablis lorsqu'ils ont été incubés avec des liposomes de kératine pendant 24 heures, comme le montre l'observation d'une surface de cheveux plus lisse en microscopie électronique. Nous avons observé que le traitement par liposomes de kératine réduisait de manière significative la dénaturation des protéines et la peroxydation des lipides dans les cheveux photo‐endommagés.

**Conclusion:**

Nous prévoyons que l'administration de kératine assistée par liposomes cationiques peut constituer une méthode efficace pour restaurer les cheveux photo‐endommagés, tant sur le plan physique que sur le plan chimique.

## INTRODUCTION

Photo‐damage caused by ultraviolet (UV) radiation from daily sunlight exposure may lead to substantial physical and chemical changes in human hair, manifesting as dryness, brittleness, roughness and colour fading [1]. Two main types of UV radiation that exert the most negative effects on human hair are UV‐A (315–400 nm) and UV‐B (280–315 nm). UV‐B breaks down the proteins in hair, especially keratin, the primary protein responsible for hair strength, by breaking down the disulphide bonds inside, causing damage to the outer layers of the hair fibres. In contrast, UV‐A primarily affects the colour and pigmentation of hair; it breaks down melanin, the natural pigment in hair, leading to colour fading, especially in lighter or chemically treated hair by deeply penetrating into the hair shaft, resulting in oxidative stress, which causes immediate damage by generating free radicals [2, 3]. Additionally, these UV radiations can initiate the oxidation of lipids in hair, which are crucial for moisture preservation and smoothness. This leads to depletion of natural oils in hair, resulting in dryness, frizz and a coarse texture, ultimately making the hair more susceptible to additional damage from mechanical stress, such as brushing [4, 5].

Keratin is a fibrous protein that constitutes approximately 80% of the hair structure, especially the cortex. Its unique composition allows it to form strong disuphide bonds, which provide mechanical strength and resilience to hair fibres [6]. Keratin‐based treatments have become more popular owing to their ability to repair damaged hair via mechanisms such as structural reinforcement by filling in gaps and defects in the hair shaft, moisture retention because of the hygroscopic nature of keratin and surface smoothing [7, 8, 9]. However, conventional keratin treatments often have limitations related to their penetration depth and duration of effect. Liposomal technology involves encapsulating active ingredients within the phospholipid bilayer or hydrophilic core, which enhances their stability and bioavailability [10]. Cationic liposomes are lipid‐based vesicles with a positive charge, allowing them to interact favourably with negatively charged hair surfaces, which not only facilitates better adhesion but also promotes deeper penetration of the encapsulated keratin [11, 12].

**FIGURE 1:**
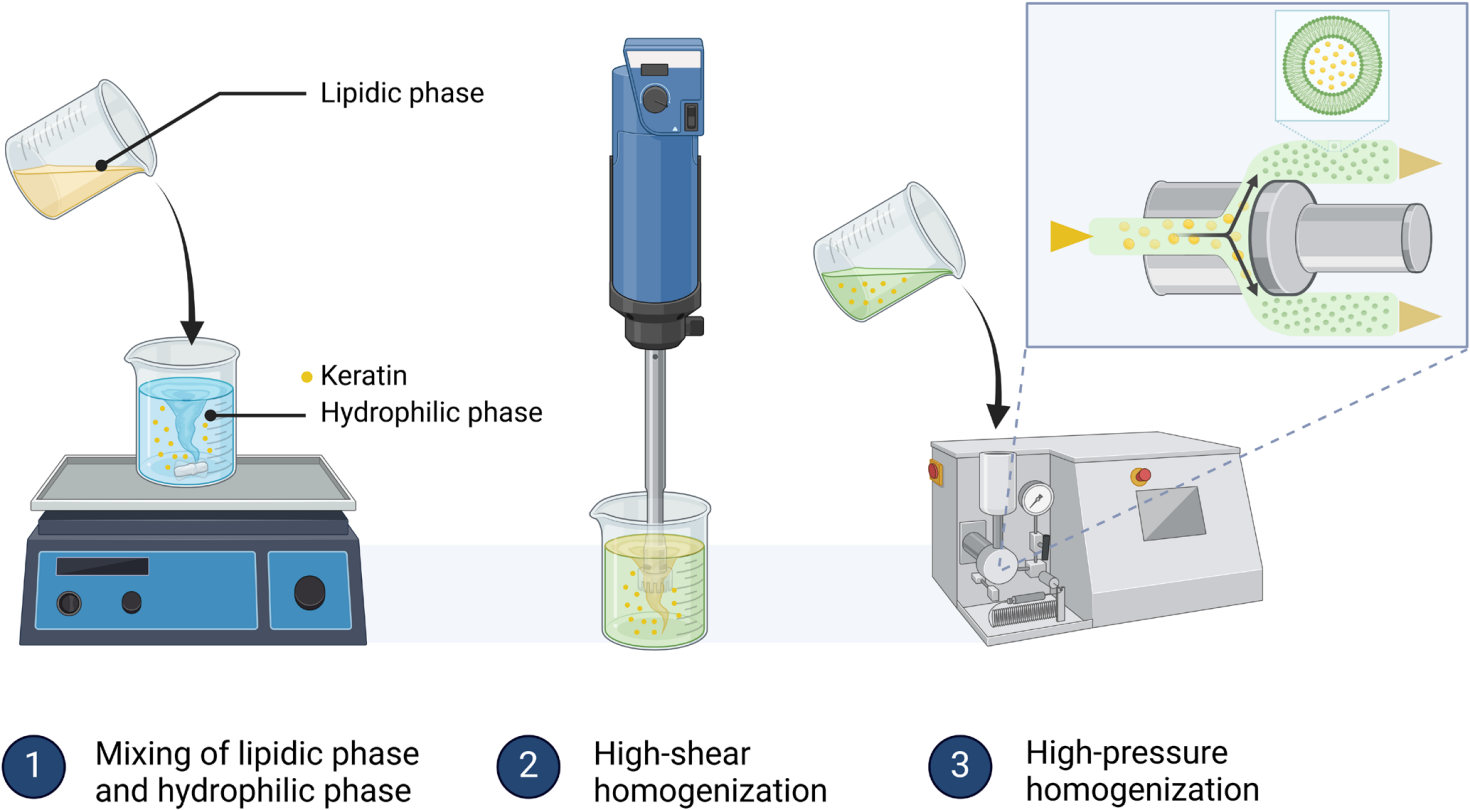
Preparation of keratin‐encapsulated cationic liposomes (KLs) via high‐pressure homogenization. In particular, a lipidic phase containing lecithin, cholesterol, ceramide NP and dipropylene glycol was heated until the mixture became miscible. Afterwards, the lipidic phase was gradually added to the hydrophilic phase containing keratin at 25°C. The mixture was subsequently homogenized for 5 min, then KLs were obtained through three cycles of 1000‐bar homogenization using a high‐pressure homogenizer. The figure was created using BioRender.com.

In this study, keratin‐encapsulated cationic liposomes (KLs) were designed to improve the adherence on the cuticle and penetration of keratin into hair fibres. KL was fabricated using the high‐pressure homogenization method (Figure 1), which provided highly stable and homogeneous liposomes. To evaluate the effect of keratin, especially KL, hair fibres were exposed to UV light before being treated with KL, followed by the examination of the hair surface and quantification of protein degradation and lipid peroxidation. Our studies show promising results of cationic liposomes as an effective carrier for hair treatment and keratin as efficacious for the restoration of photo‐damaged hair.

## MATERIALS AND METHODS

### Materials

Chitosan (degree of deacetylation ≥85%) was purchased from Chibio. Dipropylene glycol was purchased from SKC. Hydrogenated lecithin was purchased from Lipoid. Cholesterol was purchased from Active Concepts. Ceramide NP was purchased from Doosan. Hydrolysed keratin (Promois WK‐PD, MW: 400 Da) originated from sheep wool was purchased from Seiwa Kasei. Fluorescein isothiocyanate isomer I (FITC), Ellman's reagent, and L‐cysteine were purchased from Sigma‐Aldrich. EZ‐Lipid peroxidation (TBARS) assay kit was purchased from DogenBio. Bicinchoninic acid (BCA) protein assay kit was purchased from Thermo Scientific. Tissue‐Tek® Optimal cutting temperature compound (O.C.T.) was purchased from Sakura Finetek. Natural black hair tresses (Chinese female, 20–30 years old) were purchased from Japan (Beaulax Co., Ltd.). All other chemicals were of analytical grade.

### Preparation of KLs

We fabricated KLs using the high‐pressure homogenization method [10]. Briefly, the lipidic phase containing lecithin, cholesterol, ceramide NP and dipropylene glycol was heated up to 80°C until the mixture became miscible. Afterwards, the lipidic phase was gradually added to the hydrophilic phase containing 1% keratin solution in distilled water (DW) at 25°C, and the mixture was subsequently homogenized for 5 min at 2000–3000 rpm. We finally homogenized KLs using a high‐pressure homogenizer over three cycles of 1000 bars (MN400BF; Micronox). The hydrodynamic size and zeta potential of the KLs were determined using dynamic light scattering (DLS, SZ‐100; Horiba) at 25°C every week for 4 weeks.

### Characterization of KLs

The structure of KLs was analysed using cryogenic transmission electron microscopy (Cryo‐TEM, Tecnai F20; FEI) at the Korea Institute of Science and Technology (Seoul, Republic of Korea) and small angle x‐ray scattering (SAXS) at Pohang University of Science and Technology (Pohang, Republic of Korea). SAXS measurements were conducted using the 4C beamline (Pohang Light Source II, PLS II) with 3 GeV power. A light source from an in‐vacuum undulator 20 (IVU20: 1.4 m length, 20 mm period) of the Pohang Light Source II storage ring was focused with a vertical focusing toroidal mirror coated with rhodium and monochromatized with a Si(111) double‐crystal monochromator, yielding an x‐ray beam wavelength of 0.734 Å. The x‐ray beam size at the sample stage was 0.1 (V) × 0.3 (H) mm^2^. A two‐dimensional (2D) charge‐coupled detector (CCD: model Rayonix 2D SX 165, Evanston, IL, USA) was employed. The magnitude of the scattering vector, *q* = (4*π*/*λ*)sin *θ*, was 0.02 Å^−1^ < *q* < 0.25 Å^−1^, where 2*θ* is the scattering angle and *λ* is the wavelength of the x‐ray beam source. The scattering angle was calibrated with a silver behenate standard. A quartz capillary was used for loading the liquid sample. All scattering measurements were carried out at room temperature (RT). The sample‐to‐detector distance (SDD) was 1 m. Each 2D pattern was radially averaged from the beam center and normalized to the transmitted x‐ray beam intensity, which was monitored with a scintillation counter placed behind the sample. The scattering of distilled water was used as the experimental background.

### Treatment of UV‐exposed hair

We irradiated natural black hair tresses using a light source simulating UV solar irradiation (310–800 nm, 500 W m^−2^, Suntest CPS; Atlas, USA). Different exposure times, for 0 h (non‐irradiated – NI), 24 h (4272 J cm^−2^) and 48 h (4272 J cm^−2^), adjusted the UV radiation intensity. After exposure to UV light or no exposure, hair was treated with a 1% keratin solution in DW (KS) or KLs, where the hair placed in a 100Ø plate was orbitally shaken at 90 rpm for 24 h. We then washed the treated hair three times with DW (25 mL of DW each time) at 90 rpm on an orbital shaker for 15 min. We examined the surface and cross‐section of the non‐treated and treated hairs using field‐emission scanning electron microscopy (FE‐SEM) (JSM‐7900F; JEOL, Tokyo, Japan) at the Central Laboratory of Kangwon National University.

To visualize and evaluate the penetration of KS and KLs into hair fibres, we fluorescently labelled keratin with FITC. Two millilitres of 25% keratin solution were diluted in 220 mL of 60% ethanol (EtOH), 19.4 mg of FITC in 15 mL of 60% EtOH was added dropwise into the keratin solution under vigorous stirring (weight ratio of keratin: FITC = 25.77:1, molar ratio of nitrogen in keratin: FITC = 1:0.01). The labelling reaction was performed at 4°C for 24 h and the resultant solution was freeze‐dried and kept at −20°C for further use. FITC conjugation on keratin was confirmed using matrix‐assisted laser desorption/ionization time‐of‐flight mass spectrometry (MALDI‐TOF MS) with a mass range of 300–900 Da (Autoflex speed TOF/TOF; Bruker) at the Central Laboratory of Kangwon National University.

In the laboratory scale, to investigate the penetration of keratin liposome in comparison with keratin solution, we incubated the hair tresses with keratin‐FITC solution or liposomes for 1 min to 24 h. Afterwards, the hair cross‐sections were prepared by embedding in an O.C.T. solution and frozen at −20°C before being sectioned at 10 μm using a cryo‐microtome (HM525 NX; Thermo Scientific) at the Kangwon Institute of Inclusive Technology, Kangwon National University and then observed under a fluorescence microscope (DMi8; Leica Microsystems).

### Protein quantification

To extract the protein on the hair surface, 100 mg of non‐treated, KS‐ and KL‐treated hair tresses were cut to 1 cm fibres and immersed in 5 mL of DW under sonication (Branson 5510; Marshall Scientific) for 12 h at RT. The resultant solution was filtered using filter paper. Total hair‐extracted proteins were quantified using the BCA assay following the manufacturer's protocol. Briefly, 100 μL of four‐time‐diluted hair protein solution or standard was aliquoted into a 96‐well plate, 100 μL of BCA working reagent was subsequently added and reacted for 2 h at 37°C. The reactant was cooled down to RT, and the absorbance was measured at 562 nm using a plate reader. Bovine serum albumin was used as standard (conc. 0–100 μg mL^−1^).

The thiol content in the hair‐extracted protein was also determined using Ellman's assay. Briefly, 250 μL of each hair protein solution or standard was diluted in 650 μL of reaction buffer, then 100 μL of Ellman's reagent (4 mg mL^−1^ in reaction buffer) was added. The reaction was kept for 15 min at RT, then 200 μL of each reactant solution was transferred to a 96‐well plate for measuring the absorbance at 412 nm using a plate reader. L‐cysteine was used as a standard (conc. 0–0.75 μmol mL^−1^).

Total tryptophan content in UV‐exposed non‐treated, KS‐ and KL‐treated hair tresses was compared by measuring its fluorescence intensity. Briefly, 50 mg of 1‐cm hair fibres was immersed in 10 mL of 2 M NaOH solution for 48 h at RT. The resultant solution was filtered through a 0.45‐μm filter. Fluorescence intensities of three‐time‐diluted samples were measured at *λ*_ex_/*λ*_em_ = 290/345 nm using a spectrofluorophotometer (RF‐6000; Shimadzu). A higher fluorescence intensity indicated a lower degradation level of tryptophan [13].

### Lipid peroxidation measurement

Lipids from non‐treated, KS‐ and KL‐treated hair tresses were extracted by immersing 160 mg of 1 cm hair fibres in 2 mL of 2 M NaOH solution for 20 min at 80°C. The resultant solution was centrifuged at 3100 *g* for 15 min, and the supernatant was subjected to thiobarbituric acid (TBA) assay for the assessment of lipid peroxidation [14]. One hundred microliters of the supernatant containing hair‐extracted lipids was diluted with 300 μL of 9% H_3_PO_4_ and reacted with 100 μL of 30 mM TBA at 80°C for 60 min. Afterwards, 500 μL of tert‐butanol was added into each tube, followed by vortexing for 20 s and centrifugation at 3000 *g* for 20 min. Thereafter, 200 μL of the upper phase was collected into a 96‐well plate for measuring the absorbance at 532 nm using a plate reader. Malondialdehyde (MDA) was used as a standard (concentration = 0–20 μM).

### Fourier‐transform infrared spectroscopic analysis

Sulphonic acid groups (R‐SO_3_H) released due to the oxidative cleavage of S–S bonds were examined using Fourier‐transform infrared spectroscopy (FT‐IR, iS50; Thermo Fisher Scientific, MA, USA) with a built‐in attenuated total reflection (ATR, diamond crystal, 45°) mode and a deuterated triglycine sulphate (DTGS) detector at the Kangwon Radiation Convergence Research Support Center of the Korea Basic Science Institute in Kangwon National University. All spectra were collected using a 64‐scan condition in the spectral range of 4000–400 cm^−1^ at 25°C and presented without any spectral treatments except for baseline correction. For FT‐IR imaging, the hair cross‐sections were prepared by embedding the 2‐cm hair fibres in an O.C.T. solution and frozen at −20°C before sectioning at 10 μm using a cryo‐microtome. An FT‐IR microscope (Nicolet™ Continuum™, Thermo Scientific, MA, USA) equipped with a mercury‐cadmium‐telluride (MCT) detector cooled with liquid nitrogen was used to obtain all spectral images with a spatial resolution of 5 μm × 5 μm and infinity corrected visible objective (32×). All spectral images were collected in the transmission mode. The spectral images were recorded with 32 scans at 8 cm^−1^ spectral resolution.

### Statistical analysis

Statistical analysis was performed using a one‐way ANOVA in the software SigmaPlot version 14.0 with *n* = 3; error bars indicate standard deviation, unless indicated otherwise, and *p*‐values < 0.05 were considered statistically significant.

## RESULTS AND DISCUSSION

**FIGURE 2:**
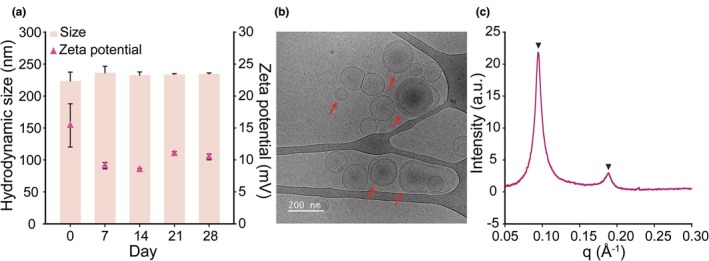
Characterization of KL. (a) Hydrodynamic size and zeta potential of KL by DLS. (b) Cryo‐TEM image of KL, scale bar = 200 nm. Arrows indicate the spots where multilamellar structures of the liposomes can be observed. (c) SAXS pattern of KL. The peaks at *q* = 0.095 and 0.190 Å^−1^ indicate the formation of bi‐ or multi‐layer structures.

KL was prepared using the high‐pressure homogenization method, as described in Figure 1 and Table 1. The synthesized KL demonstrated excellent colloidal stability over 28 days, with a nearly constant hydrodynamic size of 200–250 nm and zeta potential of 8–19 mV (Figure 2a). Previous studies reported that surface‐charged liposomes exhibited high colloidal stability for 28 days in DW at 25°C or 3 months at 4°C [10, 15]. Owing to the anionic characteristics of hair fibres, cationic chemicals are extensively utilized in conditioner formulations to improve penetration into the cortex and adherence to the hair fibres [11]. Nevertheless, the majority of previous studies have not utilized this distinctive attribute to develop a hair care product with superior penetration. This study anticipates that KL with a positive charge may penetrate more deeply into the cortical layer, which contains the highest concentration of keratin and disulphide bonds, thereby effectively facilitating the regeneration of damaged hair. The combination of the unilamellar and multilamellar structures of KL, a distinctive characteristic of liposomes produced via high‐pressure homogenization, was examined by employing cryo‐TEM (Figure 2b) [10]. Furthermore, the presence of peaks with *q* = 0.095 and 0.190 Å^−1^ on the SAXS curve of KL validated the existence of multilayered liposomes (Figure 2c), consistent with previous investigations [16, 17].

**TABLE 1:** Composition of KS and KL.

Phase	Ingredients	Composition (%)	
		KS	KL
A	Water	Up to 100.00	Up to 100.00
	Hydrolysed keratin	1.00	1.00
	Chitosan	–	0.05
B	Hydrogenated lecithin	–	0.50
	Cholesterol	–	0.25
	Ceramide NP	–	0.01
	Dipropylene glycol	–	10.00

**FIGURE 3:**
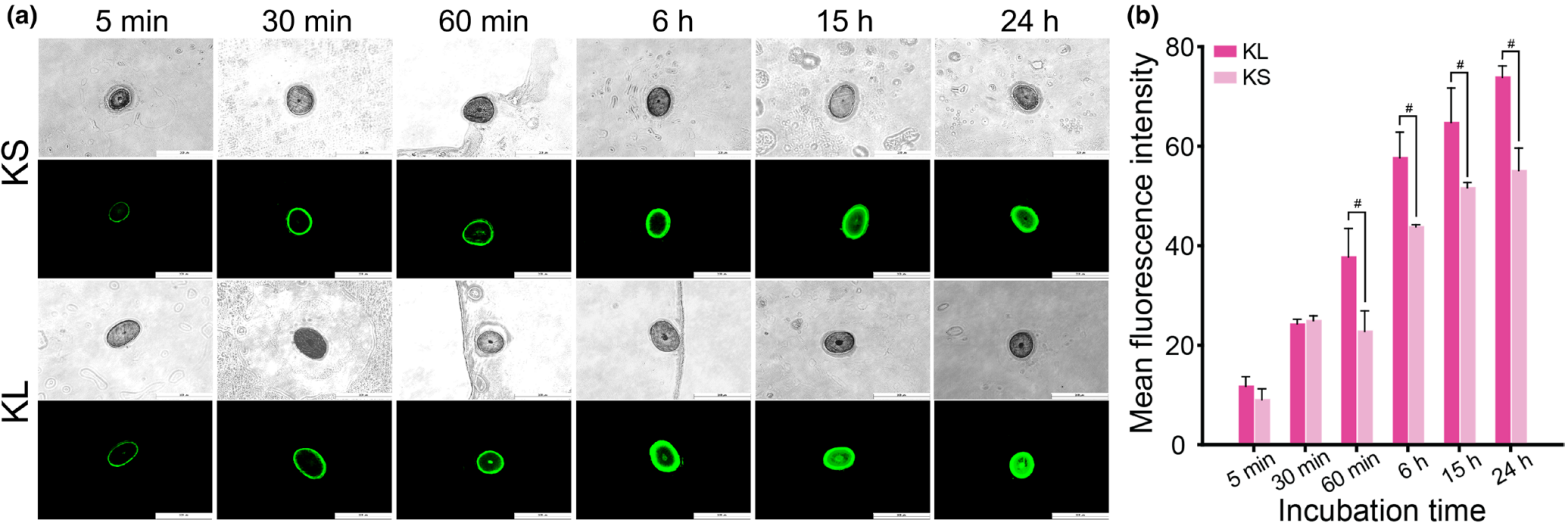
The penetration of KL and KS into hair fibre after different treatment periods. (a) Hairs were treated with KL or KS containing the same amount of fluorescently labelled keratin and incubated for the designated times, followed by observation of the cross‐sections using a fluorescence microscope. Scale bar = 200 μm. (b) Quantification of fluorescence intensities of the penetrated keratin‐FITC in the hair cross‐sections visualized by fluorescence microscopy (*n* = 3). # indicates significant differences between KL and KS‐treated samples with *p* < 0.05.

To demonstrate that cationic liposomes could enhance the penetration of keratin into the cortex layer, FITC‐labelled keratin was employed to formulate KS and KL for further visualization under a fluorescence microscope (Figure 3). Although hydrolysed keratin has a small molecular weight, which could be advantageous in permeation, the water‐based formulation of KS could not maximize the penetration in comparison with cationic liposomes. In particular, after 60 min of incubation, the hair fibre treated with KL showed significantly deeper penetration than the one treated with KS. Especially, after 24 h, only KL almost penetrated the medulla part of the hair fibre. Because the cuticle layers, the outmost layers of the hair, are hydrophobic barriers due to the presence of a compact layer of lipids (18‐methylicosanoic acid) preventing the penetration of hydrophilic molecules [11], liposomal formulation can accelerate the penetration of keratin to the cortex region of the hairs in the same way that liposomes enhance percutaneous penetration of small hydrophilic molecules across the stratum corneum [10]. Furthermore, it should also be noted that hair surfaces are slightly negatively charged because of sulphonate and carboxyl groups in the protein and lipid of hairs and more negative when the hair is damaged due to the oxidation of cysteine in the cortex and cysteine‐rich areas inside the cuticle, resulting in the release of cysteic acid [11, 18, 19]. Thus, the cationic liposomes are expected to electrostatically bind to the surface of the hairs to synergistically enhance the penetration of keratin in addition to the effects of liposomal formulation.

**FIGURE 4:**
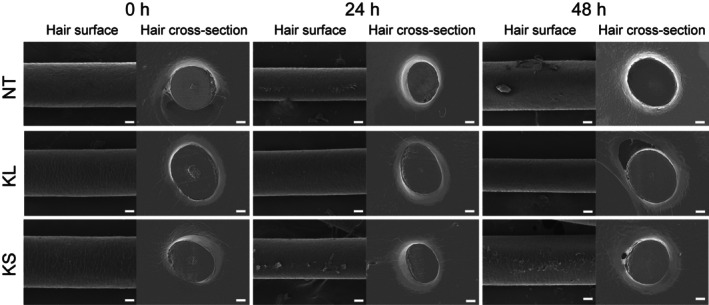
SEM images of hair fibres before and after treatment. Non‐UV‐exposed hair (0 h), 24 and 48 h‐UV‐irradiated hair tresses were treated with KL or KL for 24 h, followed by cryo‐sectioning using a microtome; the cross‐sections and the surfaces of hair samples were subsequently examined using FE‐SEM. Scale bar = 10 μm.

To evaluate the effect of keratin on the photo‐damaged hair, KS and KL were separately used to treat 24 and 48 h‐UV‐exposed hairs. The surface of the hair fibre became intensively rougher due to severe UV radiation, and the cuticle layer partially peeled off after being exposed to UV radiation for 48 h (Figure 4) [20]. Although in both KL and KS‐treated groups, the hair fibre became smoother, a significantly smooth hair surface was found only after treatment with KL, which might be due to the higher affinity of cationic material to negatively charged damaged hair [19], resulting in the higher adherence of KL to the surface of hair fibres supporting film formation, smoothing and moisture‐retaining effects of keratin [7, 8].

**FIGURE 5:**
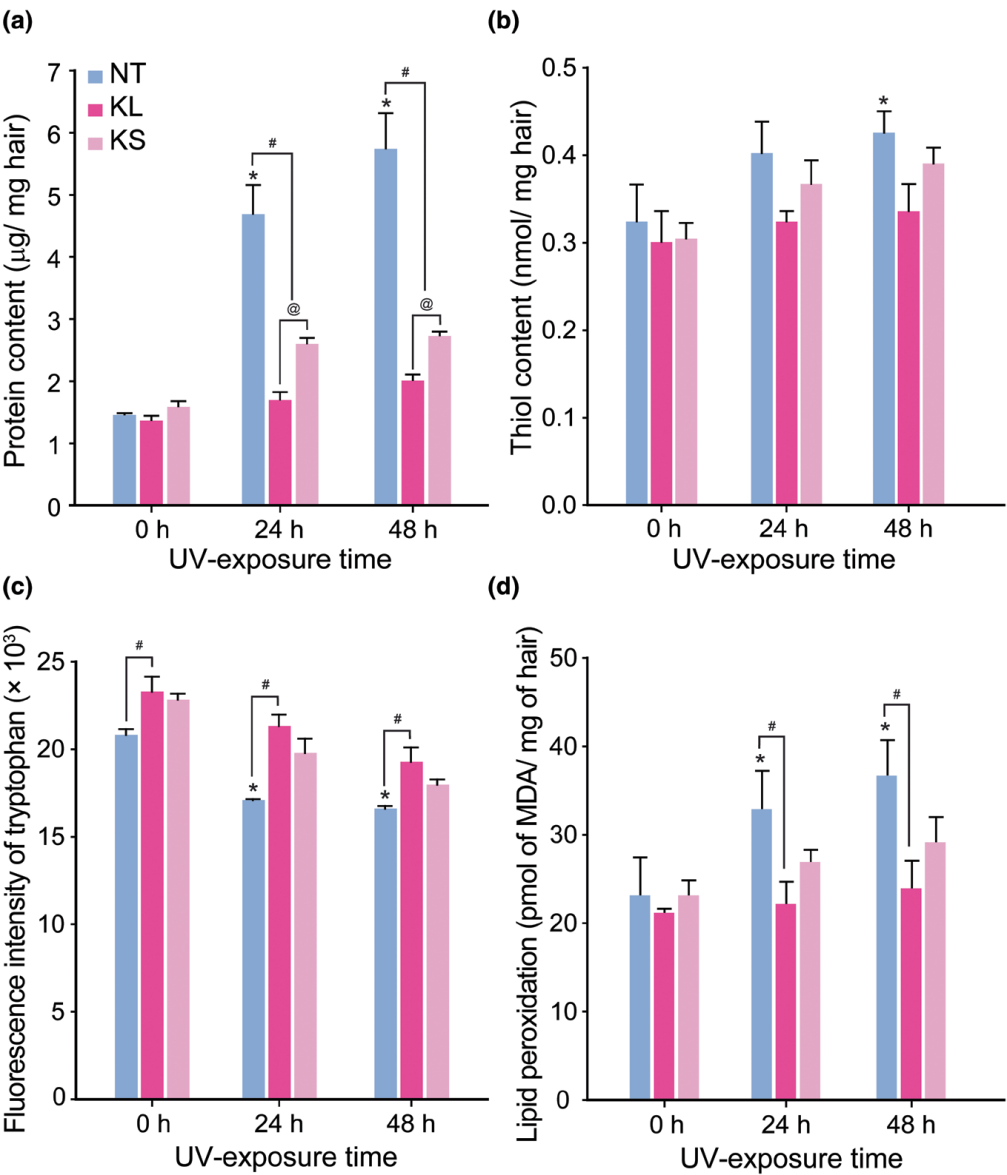
Hair recovery after treatment with keratin. (a) Protein content of non‐treated (NT), KL‐, and KS‐treated hair was extracted under a 12‐h sonication and analysed by BCA assay. (b) Thiol content in hair protein before and after treatment was determined by Ellman's assay. (c) Tryptophan content in hair fibres was quantified by measuring the tryptophan fluorescence in hair‐dissolved solution (*λ*_ex_/*λ*_em_ = 290/345 nm). (d) Hair lipid was extracted in NaOH solution; then lipid peroxides induced by UV irradiation before and after treatment were quantified using TBARS assay. * and # indicate significant differences compared with the non‐UV‐exposed hair and between KL and KS‐treated samples, respectively, with *p* < 0.05.

To evaluate the damage and recovery level of the hair after UV exposure and treatment, proteins and lipids, which are the main components of the hair structure and contribute to the integrity of the hair fibre, were quantified [20, 21]. UV radiation induced a serious loss of proteins (Figure 5a), with longer exposure times causing more severe protein degradation. In particular, NI hair only released 1.47 ± 0.02 μg protein per 1 mg hair, while 4.70 ± 0.46 and 5.75 ± 0.57 μg proteins were obtained from 24‐ and 48‐h UV‐exposed hairs, respectively. After treatment with keratin, the loss of protein was significantly reduced by approximately 64% in the case of KL‐treated hair and by 40%–50% in the case of KS‐treated hair. Owing to the superior penetration into the cortex layer, keratin did not have any influence based on the analysis of surface‐released protein, as the NI hair treated with or without KL/KS showed negligible difference. Furthermore, the protein level of KL‐treated hair nearly reached the level of the non‐irradiated hair. UV‐B is known to cause severe photo‐oxidation of amino acids and lipids, leading to the breakage of disulphide bonds between two sulphur‐containing amino acids, primarily cysteine, on the surface of the cuticle layer or inside the hair fibres. This process also results in the degradation of other amino acids, including tryptophan, and the decomposition of lipids. Consequently, this leads to an elevation in thiol content, the release of cysteic acid, a reduction in tryptophan content, and an acceleration of lipid peroxidation [2, 3, 4, 13, 19, 22]. In particular, the thiol content in the 48‐h‐irradiated hair protein increased to 0.43 ± 0.02 nmol mg^−1^ hair compared with 0.32 ± 0.04 nmol mg^−1^ hair in the NI hair protein. Due to the degradation, the fluorescence intensity of tryptophan also decreased approximately 20% in the 24‐ and 48‐h‐irradiated hair compared with that in the NI hair. Moreover, photo‐oxidation accelerated the process of lipid peroxidation, with UV‐exposed hair contributing to an approximately 1.5‐fold increase in lipid peroxidation compared with normal hair. The treatment with keratin, especially KL, could minimize the oxidative effects of hair fibres. As indicated by Ellman's assay, tryptophan fluorescence intensity analysis, and TBARS assay, thiol content and lipid peroxidation decreased while tryptophan content was enhanced in the KL group compared with those in the NI group (Figure 5b–d).

**FIGURE 6:**
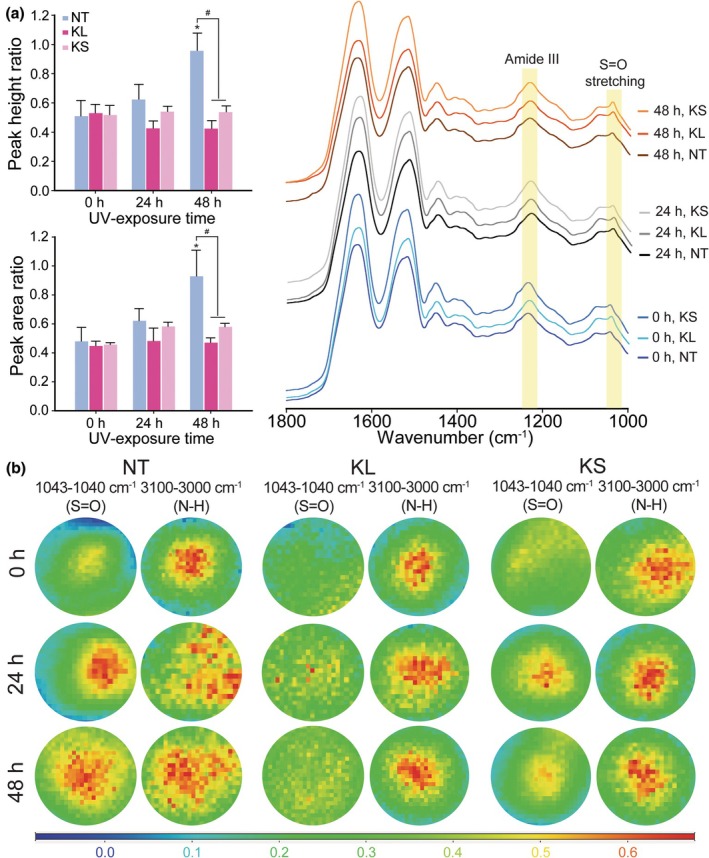
FT‐IR analysis of S=O from sulphonic acid group due to the oxidation of disulphide bonds caused by UV radiation. (a) Peak height and peak area ratio of S=O and amide III (as internal standard) based on FT‐IR spectra. (b) FT‐IR microscopy of the hair cross‐sections. The cryo‐sections of the hair were visualized at 10X magnification using a microscope equipped with a mercury‐cadmium‐telluride (MCT) detector. The spectral images were recorded with 32 scans at 8 cm^−1^ spectral resolution. The intensity scale bar below the images indicates the intensity of each chemical group in an arbitrary unit. N–H is used as an internal standard and marks the position of hair cross‐sections. All hair samples had strong intensities of N–H, while the intensities of S=O groups were significantly different due to the different oxidation and recovery levels. * and # indicate significant differences compared with non‐UV‐exposed hair and between KL and KS‐treated samples, respectively, with *p* < 0.05.

The vibrational modes of hair fibre components before and after keratin treatment were studied using FT‐IR spectroscopy and IR spectroscopic imaging (Figure 6). The intensity of the S=O group increased in the 48‐h UV‐exposed hair, which was quantified based on the peak area and peak height ratio of amide III [23, 24]. Similar results were observed in the IR images, where red and yellow areas dominated in the 48‐h UV‐exposed hair fibre without treatment. The KS‐ and KL‐treated hair fibres exhibited a substantial reduction of S=O intensities, as observed in both the FT‐IR spectra and images, while the IR images of all hair samples exhibited similar intensities of N‐H moieties, which were used as the control [25]. Taken together with the results of protein and lipid peroxide content, we can speculate that keratin has a significant effect on the recovery of photo‐damaged hair. Moreover, keratin encapsulated in cationic liposomes could further enhance the penetration of keratin into hair fibres, resulting in better hair repair.

## CONCLUSIONS

In this study, we successfully fabricated highly stable cationic liposomes encapsulated with keratin using high‐pressure homogenization, which supported better penetration of keratin into hair fibre compared with the solution form. Both 24‐ and 48‐h UV‐exposed hair exhibited enhanced recovery after a 24‐h treatment with keratin, especially keratin liposomes, as demonstrated by the smoother surface appearance of the hair fibre and chemical contents, namely hair proteins and lipids. Therefore, our designed KLs can be considered a potential material for leave‐on hair care products such as daily hair serum or essence for repairing photo‐damaged hair.

## CONFLICT OF INTEREST STATEMENT

These authors do not claim any conflict of interest regarding this study.
